# Phylogeography of *Eomecon chionantha* in subtropical China: the dual roles of the Nanling Mountains as a glacial refugium and a dispersal corridor

**DOI:** 10.1186/s12862-017-1093-x

**Published:** 2018-02-09

**Authors:** Shuang Tian, Yixuan Kou, Zhirong Zhang, Lin Yuan, Derong Li, Jordi López-Pujol, Dengmei Fan, Zhiyong Zhang

**Affiliations:** 1 Laboratory of Subtropical Biodiversity, Jiangxi Agricultural University, Nanchang, Jiangxi 330045 China; 2 Jingdezhen University, Jingdezhen, Jiangxi 333000 China; 3 Germplasm Bank of Wild Species in Southwest China, Kunming Institute of Botany, Chinese Academy of Sciences, Kunming, Yunnan 650204 China; 4 Instituto Botánico de Barcelona, IBB, CSIC-ICUB, Passeig del Migdia s/n, 08038 Barcelona, Spain

**Keywords:** Phylogeographic structure, Chloroplast intergenic spacer, Ribosomal internal transcribed spacer, Microsatellite, Dispersal corridor, *Eomecon chionantha* Hance

## Abstract

**Background:**

Mountains have not only provided refuge for species, but also offered dispersal corridors during the Neogene and Quaternary global climate changes. Compared with a plethora of studies on the refuge role of China’s mountain ranges, their dispersal corridor role has received little attention in plant phylogeographic studies. Using phylogeographic data of *Eomecon chionantha* Hance (Papaveraceae), this study explicitly tested whether the Nanling Mountains, which spans from west to east for more than 1000 km in subtropical China, could have functioned as a dispersal corridor during the late Quaternary in addition to a glacial refugium.

**Results:**

Our analyses revealed a range-wide lack of phylogeographic structure in *E. chionantha* across three kinds of molecular markers [two chloroplast intergenic spacers, nuclear ribosomal internal transcribed spacer (nrITS), and six nuclear microsatellite loci]. Demographic inferences based on chloroplast and nrITS sequences indicated that *E. chionantha* could have experienced a strong postglacial range expansion between 6000 and 1000 years ago. Species distribution modelling showed that the Nanling Mountains and the eastern Yungui Plateau were the glacial refugia of *E. chionantha*. Reconstruction of dispersal corridors indicated that the Nanling Mountains also have acted as a corridor of population connectivity for *E. chionantha* during the late Quaternary.

**Conclusions:**

Our results suggest that the Nanling Mountains may acted dual roles as a dispersal corridor in east-west direction and as a glacial refugium in subtropical China during the late Quaternary. The population connectivity mediated by the mountain range and a strong postglacial range expansion are the most likely reasons for the lack of phylogeographic structure in *E. chionantha.* The hypothesis of dual roles of the mountain range presented here sheds new insights into the phylogeographic patterns of organisms in subtropical China.

**Electronic supplementary material:**

The online version of this article (10.1186/s12862-017-1093-x) contains supplementary material, which is available to authorized users.

## Background

Given their extremely diverse topography and a wide spectrum of environmental conditions within short distances, mountains have generally provided refuge for species, especially during the Neogene and Quaternary global climate changes [1–3]. In addition, mountains have often offered dispersal corridors, allowing for range expansions/contractions, population connection, and gene flow between otherwise fragmented populations [1, 4, 5]. Both functions of refuge and dispersal corridor are extremely important for the persistence of organisms during periods of large climate change such as the late Cenzoic [6]. For example, many mid-elevation mountain ranges and hilly areas in southern and central Europe did not only provide shelters for the survival of *Fagus sylvatica* during the Quaternary glacial periods, but also facilitate the northward spread of the residual populations during inter−/postglacial periods, because beeches found their humid habitats that were absent on the great plains [7]. Such cases were also reported in the Korean Peninsula of east Asia. The uninterrupted Baekdudaegan Mountains, that runs from north to south over 1600 km, acted as a true dispersal corridor for a large assemblage of boreal and temperate elements during the Quaternary climate changes beyond serving as a refugium for many species [5].

Harboring more than 30,000 vascular plants, China is one of 17 world’s megadiversity countries [8]. Many Tertiary lineages (such as *Cathaya*, *Ginkgo*, *Glyptostrobus*, *Metasequoia*, and so forth) that were once widely distributed along the Northern Hemisphere are today endemic to China [9, 10]. An important reason is that a high percentage of the mountainous lands in the country (in China over 50% is mountainous [11]) served as refuge areas for those ancient lineages [2, 10, 12–15]. During the past decade, increasing molecular phylogeographic studies on Chinese plant species showed that climatic refugia identified by DNA-based data (reviewed in [16, 17]) are largely congruent with the centers of endemism [15], highlighting the sheltering function of China’s mountain ranges. However, compared with numerous studies on the refuge role of China’s mountain ranges, their function as dispersal corridors has received much less attention (but see [18, 19]). In addition, the mountain corridors in China have been mostly inferred subjectively according to the distribution patterns of certain taxa or genetic lineages, while very few have been tested explicitly using reproducible methods (but see [20]).

**Fig. 1:**
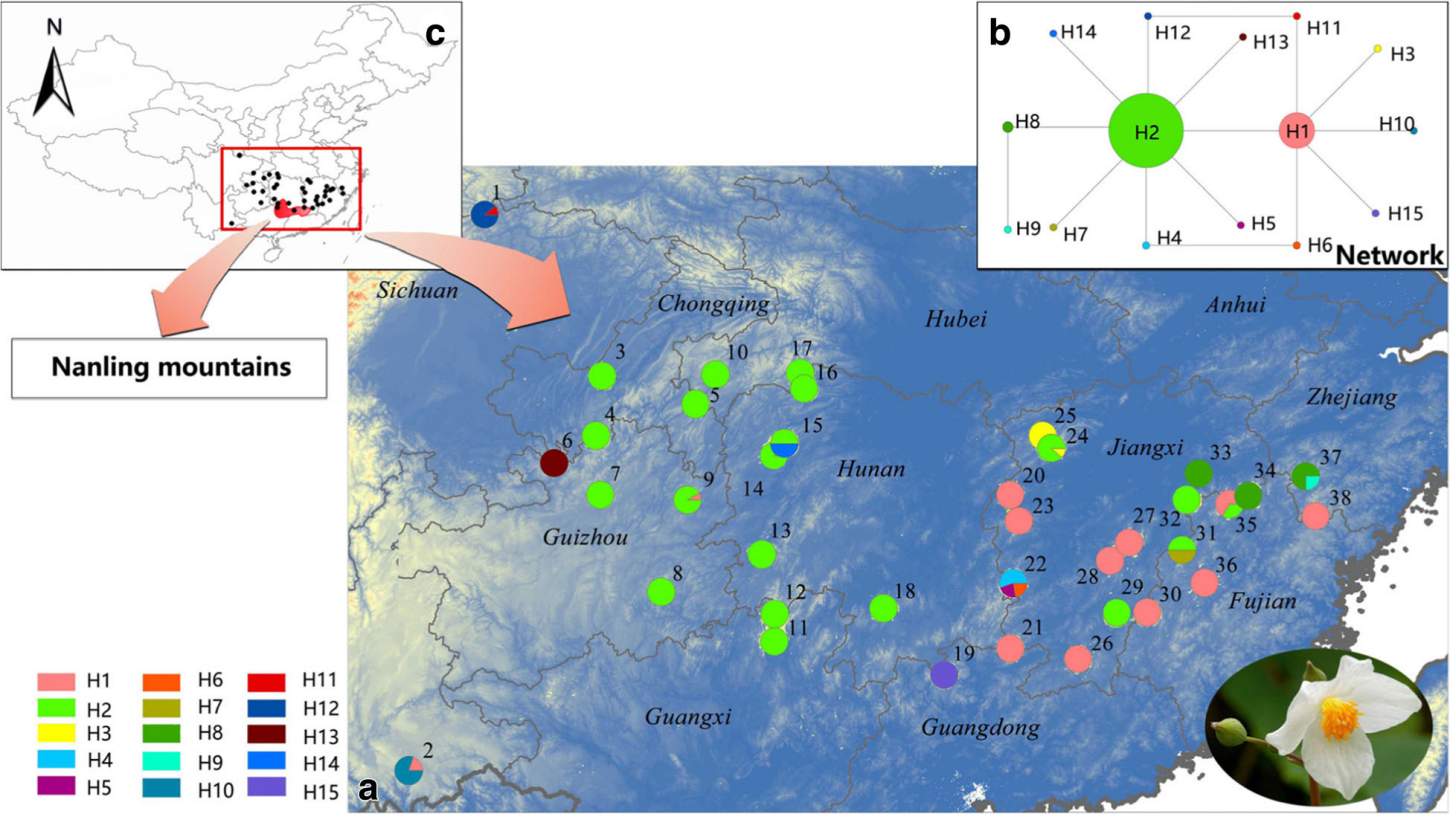
**a** Distribution of chloroplast haplotypes of *Eomecon chionantha.* **b** The network of 15 chloroplast haplotypes. **c** The distribution of *Eomecon chionantha* in China, the Nanling mountains is indicated with dark red color

The Nanling Mountains sensu *lato* (i.e., including the mountains on the Guangxi-Guangdong and Hunan-Jiangxi borders) is a series of five connected mountain ranges that spans from west to east for more than 1000 km (Fig. 1c): Yuechengling, Dupangling, Mengzhuling, Qitianling and Dayuling mountains. Also known as the “Five Mountains”, and though not very high (average about 1000 m), the Nanling range is a natural dividing line in subtropical China, effectively separating the Yangtze (Changjiang) River drainage area (characterized by a central or north-subtropical climate) from the Pearl (Zhujiang) River drainage area (of south-subtropical or tropical climate). The Nanling Mountains is one of the key biodiversity hotspots in China [14, 21] and has long been viewed as a major glacial refugium [12, 16, 17] as well as an important ‘ice-free’ corridor in east-west direction [16, 18]. While its role as a glacial refugium has been demonstrated by different studies, robust evidence for the role of a corridor is still lacking.

*Eomecon chionantha* Hance is a perennial herb (10–30 cm in height) of the monotypic genus *Eomecon* from the poppy family (Papaveraceae, [22]). The Chinese name *xueshuicao* means ‘bleeding herb’ because its leaves and rhizomes ooze orange-red sap when bruised or cut. It is an entomophilous species (with bees and flies being the most efficient pollinators) that has mixed-mating system [23]. *E. chionantha* is a rhizomatous plant that also produces subterranean runners, which allows vegetative propagation. It is a potentially important economic plant given the molluscicidal activity of its alkaloids (they have shown to be active against *Oncomelania hupensis*, the only intermediate host of *Schistosoma japonicum* in China; [24]). *E. chionantha* always grows in shady and moist habitats along streams or trails in forests or at forest margins in mountainous areas (300–2000 m; [22]). The distribution of *E. chionantha* confines to subtropical China, where plenty of precipitation (800–1600 mm/year) is brought by the East Asian monsoon and Indian Monsoon. The Nanling Mountains are the southern limit of its distribution [12].

Such a distribution pattern and the close association with mountainous forests makes *Eomecon* a good case study to test whether the Nanling Mountains were both a major refugium and a mountain corridor for the persistence and connectivity of plant populations in subtropical China. Phylogeography, particularly in combination with species distribution modelling (SDM) and GIS-based methods, has been successfully used in locating glacial refugia and migratory corridors during the past decades [25, 26]. In this study, we investigated the phylogeographic pattern of *E. chionantha* using multi-loci genetic data, and then hindcasted the distribution at the Last Glacial Maximum (LGM) and Mid-Holocene (MH). At last, a recently developed method that integrates genetic and geospatial data (the categorical least cost path, CLCP [25]) was used to visualize the population connectivity of *E. chionantha*.

## Methods

### Population sampling and molecular procedure

Thirty-eight populations were collected after extensive field surveys throughout subtropical China from 2010 to 2014 (Table 1). Because this herb can spread through rhizomes to form patches, sampled individuals were spaced by ca. 20–50 m to avoid repeatedly collecting the same individual (i.e., ramets belonging to the same genet) except for a few small populations. All samples were desiccated in silica gel and stored at −20 °C until being processed. Genomic DNA was extracted using a modified CTAB procedure [27].

**Table 1:** Sample location, sample size, and genetic variation of 38 populations of *Eomecon chionantha*

Province	Location	Code	Lat. (°N)	Long. (°E)	Alt. (m)	*N* cp/ITS/nSSRs	cp	ITS	nSSRs			
							*h*_S_	*h*_S_	*A*_R_	*H*_E_	*H*_O_	*F*_IS_
Gansu	Wenxian	1	32.7142	105.2310	860	9/7/9	0.2222	0.4762	3.50	0.593	0.500	0.213
Yunnan	Wenshan	2	23.3789	103.9597	2049	10/13/15	0.3556	0.7821	2.48	0.396	0.522	−0.289
Chong qing	Jinfoshan	3	29.0529	107.1999	1253	9/3/14	0	0	2.33	0.412	0.510	−0.201
	Jinfoshan	4	28.9917	107.0978	1388	3/0/3	0	–	1.17	0.083	0.167	−1.000
	Chongqing	5	29.5300	108.7700	456	4/1/10	0	–	5.83	0.689	0.666	0.092
Guizhou	Xishui	6	28.5374	106.3943	1021	10/0/15	0	–	2.00	0.500	0.989	−0.978
	Suiyang	7	28.2842	107.1716	1540	10/9/15	0	0	4.99	0.580	0.600	0
	Leigongshan	8	26.3703	108.1953	1464	14/13/12	0	0.6667	6.50	0.746	0.875	−0.131
	Fanjingshan	9	27.9153	108.6358	1323	12/6/15	0.1667	0	8.24	0.805	0.818	0.019
Hubei	Xingdoushan	10	30.0256	109.1020	828	9/0/15	0	–	5.64	0.690	0.611	0.148
Guangxi	Lingui	11	25.5356	110.0884	321	9/18/12	0	0.5294	3.00	0.485	0.558	−0.106
Hunan	Suining	12	26.3835	110.0971	882	10/10/12	0	0	4.00	0.613	0.447	0.311
	Huitong	13	26.6144	109.8807	564	10/18/15	0	0.5294	2.16	0.510	0.989	−0.935
	Guzhang	14	28.6600	110.0824	1390	10/12/15	0.2000	0.3030	7.11	0.784	0.742	0.090
	Xiaoxi	15	28.8675	110.2579	660	10/7/14	0.5556	0	3.64	0.533	0.591	−0.068
	Sangzhi	16	29.7871	110.0918	1390	10/1/0	0	–	–	–	–	–
	Shimen	17	30.0496	110.5237	1830	10/12/0	0	0.5455	–	–	–	–
	Yangmingshan	18	26.0932	111.9219	867	10/20/15	0	0.5263	2.82	0.429	0.555	−0.259
	Yizhang	19	24.9719	112.9421	812	10/16/14	0	0.5333	4.61	0.605	0.690	−0.106
	Daweishan	20	28.4275	114.0516	412	8/10/0	0	0.4667	–	–	–	–
Jiangxi	Dayu	21	25.4168	114.0499	520	3/6/3	0	0.6000	2.17	0.389	0.667	−0.600
	Jinggangshan	22	26.5175	114.0994	896	9/0/15	0.6667	–	4.52	0.547	0.530	0.065
	Luxi	23	27.5613	114.1933	525	11/7/5	0	0	2.67	0.499	0.633	−0.160
	Xiushui	24	28.7871	114.7388	651	9/7/15	0.2222	0.5714	3.29	0.411	0.579	−0.377
	Guanshan	25	28.5549	114.5923	488	10/10/15	0	0.5556	5.02	0.630	0.733	−0.130
	Xinfeng	26	25.2429	115.1833	278	9/20/15	0	0.5263	1.50	0.250	0.500	−1.000
	Yihuang	27	27.3806	116.0814	500	4/9/5	0	0	1.67	0.267	0.444	−0.569
	Yifeng	28	26.8678	115.7725	470	10/10/5	0	0	3.17	0.457	0.500	0.016
	Ruijin	29	26.0231	115.8370	320	4/6/6	0	0	1.67	0.275	0.528	−0.900
	Shicheng	30	26.0245	116.3417	407	8/16/6	0	0.5333	3.00	0.514	0.694	−0.269
	Lichuan	31	27.0694	116.9326	682	10/0/15	0.5556	–	4.30	0.572	0.646	−0.095
	Bijiafeng	32	27.9242	117.8781	470	6/15/11	0	0.4762	3.67	0.570	0.476	0.231
	Guixi	33	28.3597	117.2083	1100	8/10/9	0	0	2.33	0.347	0.375	−0.015
	Shangrao	34	27.9864	118.0458	997	8/4/15	0	0.5000	3.49	0.544	0.481	0.153
	Wuyishan	35	27.8433	117.7264	909	3/0/14	0.6667	–	4.95	0.644	0.553	0.203
Fujian	Jiangle	36	26.5196	117.2682	699	9/0/15	0	–	3.48	0.515	0.522	0.024
Zhejiang	Suichang	37	28.3159	119.0049	586	8/3/0	0.4286	0	–	–	–	–
	Qingyuan	38	27.6464	119.1687	586	7/2/0	0	0	–	–	–	–
Total/mean		38					0.5662	0.7830	3.66	0.512	0.597	−0.201

-: data not available

Two intergenic spacers (IGSs) of the chloroplast genome, *rbc*L-*atp*B [28] and *trn*C-*rpo*B [29], and nuclear ribosomal internal transcribed spacer (nrITS) [30] were amplified and sequenced. The PCR primers of the three genomic fragments are listed in Additional file 1. Amplification reactions were carried out in a volume of 20 μl, containing 10 μl 2 × Taq PCR MasterMix (Tiangen, Shanghai, China), 1 μl each forward and reverse primer (0.2 uM), 1 μl template DNA (ca. 50–100 ng) and 7 μl ddH_2_O. Amplification was carried out in a Bioer XP cycler (Bioer, Hangzhou, China) programmed for an initial step of 240 s at 94 °C, followed by 30 cycles of 60 s at 94 °C, 60 s at 50 °C (*rbc*L-*atp*B), 50 °C (*trn*C-*rpo*B), or 53 °C (nrITS), 60 s at 72 °C, and a final step of 600 s at 72 °C. Sequencing reactions were conducted with the corresponding forward and reverse primers commercially by Sangon Biotech Co., Ltd. (Shanghai, China). Six nuclear microsatellite (or nuclear simple sequence repeat, nSSR) loci (XSCO35, XSCO37, XSCO78, XSCQ13, XSCQ25, XSCQ60) that were specifically developed for *E. chionantha* were amplified and genotyped (primers and the PCR procedure were detailed in [31]). The PCR products were then separated on ABI 3730xl DNA Analyzer (Applied Biosystems, USA) to determine the precise fragment sizes at each of the six loci.

### Phylogeographic and population genetic analyses

All sequences of chloroplast IGSs and nrITS were edited with Sequencher (GeneCodes Corporation, Ann Arbor, MI, USA) and were aligned using Clustal_X v1.81 [32]. Indels were discarded in subsequent analyses. Haplotype relationships were assessed using the median-joining network method [33] in Network v4.1.0.8 (http://www.fluxus-engineering.com). We used the program Haplonst [34] to calculate the total and within-population haplotype diversity (*h*_T_ and *h*_S_), as well as population differentiation based on ordered (*N*_ST_) and unordered (*G*_ST_) haplotypes. The values of *N*_ST_ and *G*_ST_ were then compared using *U*-statistics to test for the presence of phylogeographic structure.

For the nSSR dataset, all six loci were checked for possible null alleles using Micro-checker v2.2.3 [35]. Deviations from Hardy-Weinberg Equilibrium (HWE) were performed with ARLEQUIN using an exact test [36] with 10,000 dememorization steps at species and population levels. Linkage disequilibrium (LD) among loci was tested using FSTAT v2.9.3 [37]. Statistical significance (α = 0.05) for inferring LD or departures from HWE was evaluated based on 1000 permutations, and corrected for multiple tests using the sequential Bonferroni method [38]. Genetic diversity was measured by allelic richness (*AR*, [39]), observed heterozygosity (*H*_O_), and expected heterozygosity (*H*_E_, [40]).

Genetic subgroups in the nSSR dataset were identified by a Bayesian analysis in STRUCTURE v2.3.1 [41] using the admixture model and assuming independent allele frequencies among populations. The number of clusters (*K*) was set to vary from one to 33. For each value of *K*, we performed 10 runs with a burn-in length of 10,000 and a run length of 100,000 Markov chain Monte Carlo (MCMC) replications. The mean log-likelihood for each value of *K*, [ln Pr(X|K)], and Δ*K* were employed to identify the most likely number of genetic clusters [42].

Because our STRUCTURE analysis did not included geographical information for the inference of population structure, we used a modified algorithm that includes spatially explicit prior distributions describing which sets of individuals are likely to have similar cluster membership [43]. In this approach, implemented in the program TESS v2.3 [44, 45], clusters corresponding to spatially and genetically continuous units separated by small discontinuities where genetic barriers are crossed were identified. We ran TESS with an admixture analysis for *K*max ranging from 2 to 9 (10 replicates for each value), with a burn-in period of length 20,000 cycles and estimation of length 30,000 additional cycles. For each run, we computed the Deviance Information Criterion (DIC), a model-complexity-penalized measure of how well the model fits the data. The smallest DIC values were obtained for the maximum number of clusters that best suits the data. We averaged the estimated admixture coefficients (Q matrix) over the ten runs with the smallest values of the DIC using the software Microsoft Office Excel 2007.

### Demographic inference

We calculated the changes in effective population size for chloroplast and nuclear sequences separately by means of Bayesian skyline plots (BSP) in Beast v1.8.2 [46]. The combined analysis of chloroplast and nuclear sequences using the extended Bayesian skyline plot [47] was not applicable because the effective sample sizes (ESS) of the analysis were low (<100). MCMC chains were run for 10,000,000 generations for chloroplast sequences under GTR + G model and nrITS sequences under the HKY + G model chosen by jModelTest v2.1.5 [48]. The substitution rate of nrITS sequences (4.16 × 10^−9^ s/s/y) reported for *Meconopsis* of the Papaveraceae family [49] was used to date the demographic events. The synonymous substitution rate of sugarcane, maize and rice chloroplast genomes (1.52 × 10^−9^ s/s/y) was adopted for the two chloroplast IGSs [50].

### Species distribution modelling and population connectivity

Assuming the species has not changed its climatic preference through time (i.e., niche conservatism), we reconstructed the potential range of *E. chionantha* at the LGM, MH and the present time according to its current distribution using a maximum entropy model (Maxent v3.1.0, [51]). The current distribution information for *E. chionantha* was obtained from collection records from the Chinese Virtual Herbarium (CVH, http://www.cvh.ac.cn/) combined with the sampling sites in this study. A total of 87 presence records were used for SDM construction. Current (1950–2000) and past [LGM ~ 21,000 yr. BP and MH ~6000 yr. BP] bioclimatic variables were downloaded from the WorldClim database (http://www.worldclim.org/) at 2.5-arcmin resolution. We used LGM and MH data simulated by the Community Climate System Model (CCSM; [52]). To aviod over-fitting of niche models, 6 variables with pairwise Pearson correlation coefficients *r* ≤ 0.5 (BIO1, Annual Mean Temperature; BIO3, Isothermality; BIO4, Temperature Seasonality; BIO7, Temperature Annual Range; BIO8, Mean Temperature of Wettest Quarter; BIO14, Precipitation of Driest Month) were retained for SDM analyses. Each model was run 10 times using the default parameters (convergence threshold of 10^−5^, maximum iterations of 500 and regularization multiplier of 1) and the following user-selected features: application of a random seed, duplicate presence records removal and logistic probabilities used for the output. Eighty percent of species records were used to train the model and 20% to test the model.

Putative dispersal corridors for population connectivity in *E. chionantha* were constructed by combining haplotype or ribotype networks and SDMs. We inverted the SDM to a “dispersal cost” layer (i.e., high probability of occurrence in the SDM has a low cost to dispersal through that region). Then, we generated a population connectivity map by summing the least cost paths (LCPs) among all shared and sister haplotypes or ribotypes from different populations in ArcGIS v9.3 (ESRI, Redlands, CA, USA) using the dispersal cost as the friction layer. The LCPs were classified into three categories: the lowest 1% LCPs, the lowest 2% LCPs, the lowest 5% LCPs and then subsequently summed each pairwise comparison [25]. Areas with the hottest colors are those hypothesized to offer the greatest ease of dispersal.

## Results

### Chloroplast DNA diversity and phylogeographic structure

The aligned length of *E. chionantha* was 796 bp for *rbc*L-*atp*B and 1142 bp for *trn*C-*rpo*B. Three and 10 substitutions were found in *rbc*L-*atp*B (GenBank accession numbers: KP658781-KP658787) and *trn*C-*rpo*B (GenBank accession numbers: KP6587811-KP658824), respectively. Six indels (4–20 bp) were discarded in subsequent analyses, resulting in a reduced length of 1876 bp of the pooled chloroplast sequences. Fifteen chloroplast haplotypes (chlorotype H1–15) were identified in 38 populations. At the species level, the cpDNA data revealed moderate haplotype diversity (*h*_T_ = 0.566). Haplotype diversity at population level varied from 0 to 0.667, with the highest occurring in populations 22 and 35 (Table 1).

The chlorotype network revealed that most haplotypes were separated by one mutation to the two most common haplotypes, H1 and H2 (Fig. 1b); the only exception was H9, that was derived from H8 by one step. H1 occurred exclusively in east populations (10 populations), except for a southwest population (population 2, P2) and a west population (P9). H2 was more widespread (20 populations), but with higher frequencies in the species’ western range. Up to 10 haplotypes were exclusive to a single population, whereas one (H3) was shared by two populations (P24 and P25) and one (H8) by three populations (P33, P34 and P37) (Fig. 1a). Phylogeographic structure tested by Haplonst was not obvious in *E. chionantha*, because *N*_ST_ (0.792) was not significantly larger than *G*_ST_ (0.780) (*U* = 1.64, *P* < 0.05).

### nrITS diversity and phylogeographic structure

A total of 301 nrITS sequences across 213 individuals from 31 populations were obtained. The aligned length was 725 bp with 25 nucleotide substitutions and two indels (that were discarded subsequently). Fifteen nuclear ribosomal ITS types (ribotypes, R1-R15) were identified among all samples surveyed (GenBank accession numbers: KP658788-KP658802).

**Fig. 2:**
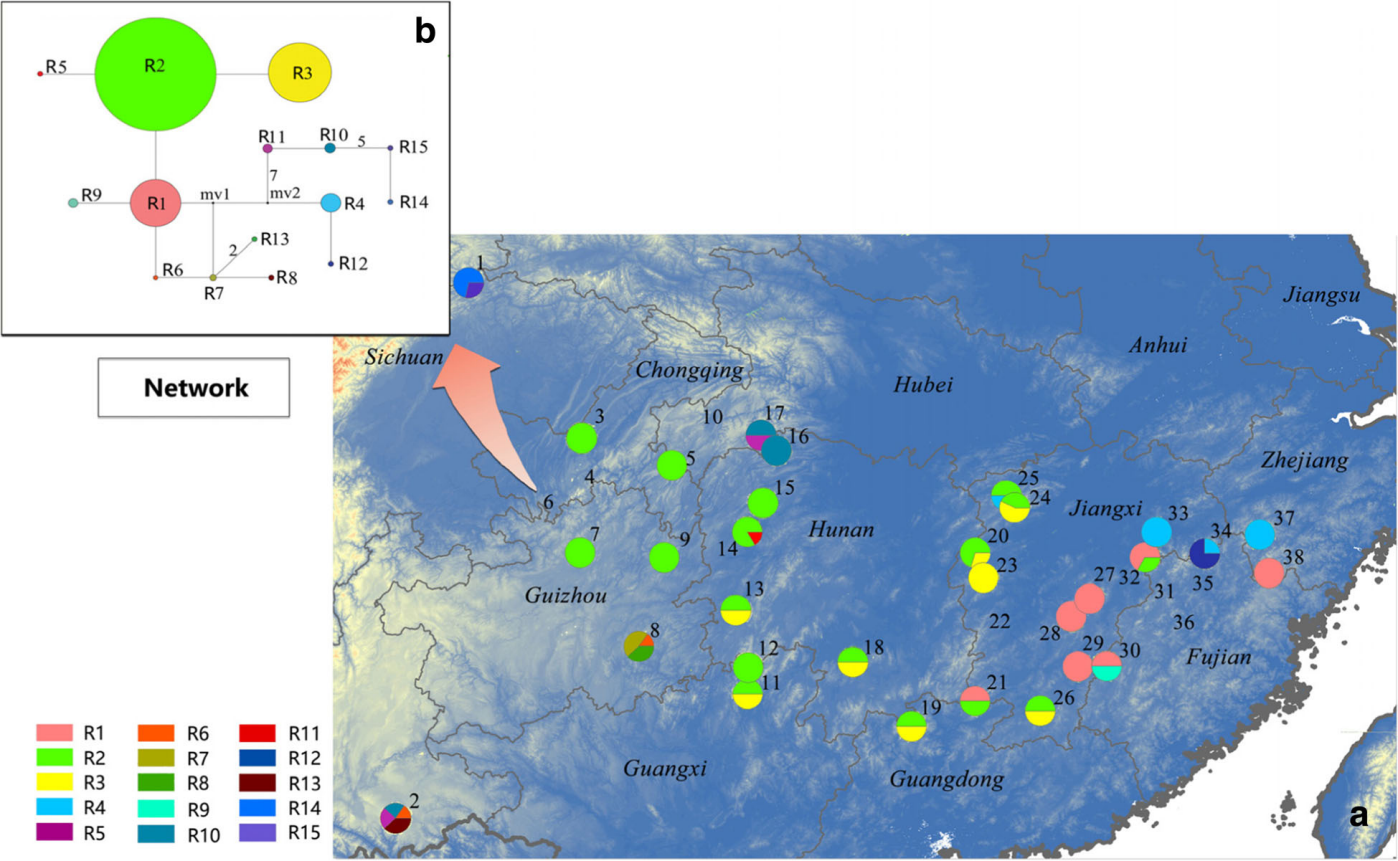
**a** Distribution of ITS ribotypes of *Eomecon chionantha.* **b** The network of 15 ribotypes

The network of 15 ribotypes was rather complex, without any obviously differentiated clade (Fig. 2b). Three most common ribotypes (R1, R2 and R3) were successively connected by one mutation. The most common ribotype (R2) occurred across the whole range of *E. chionantha*, being only absent in 14 populations (45.2%). The second most common ribotype (R3) occurred mainly in central populations, whereas the third most common (R1) largely occurred in the eastern populations. The remaining ribotypes were either population-specific or regionally restricted (Fig. 2a). Phylogeographic structure was not significant at the nrITS locus (*N*_ST_ = 0.719, *G*_ST_ = 0.635, *U* = 0.82, *P* < 0.05).

### Nuclear microsatellite diversity and population structure

Using Micro-checker, the frequency of the possible null alleles at each of the six loci surveyed was found to be lower than the threshold frequency (v = 0.12) across 389 individuals belonging to the 33 populations that were successfully genotyped. There was no evidence for LD. None of the six loci deviated from HWE significantly when all samples were treated as a single population.

A total of 117 alleles were detected at the six loci resolved for *E. chionantha*. The observed alleles per locus ranged from three to 15, and the alleles per locus across populations ranged from 1.17 to 8.64 (data not shown). Within-population allelic richness (*A*_R_) and expected heterozygosity (*H*_E_) ranged from 1.10 to 1.83 and from 0.083 to 0.805, respectively. Regression analyses showed that either *A*_R_ or *H*_E_ was dependent on latitude or longitude (*P* = 0.16).

Genetic differentiation at six microsatellite loci was moderate (*G*_ST_ = 0.367). In the Bayesian analysis of population structure, we were not able to find a meaningful *K* value, indicating there is no evidence of genetic cluster in *E. chionantha* (Additional file 2). For TESS analyses on SSR data, although DIC values were the smallest when *K*max = 6 (Additional file 2), the clusters had not geographical association. Therefore, TESS did not find any geographically meaningful genetic cluster either.

### Demographic history based on cpDNA and nrITS sequence data

**Fig. 3:**
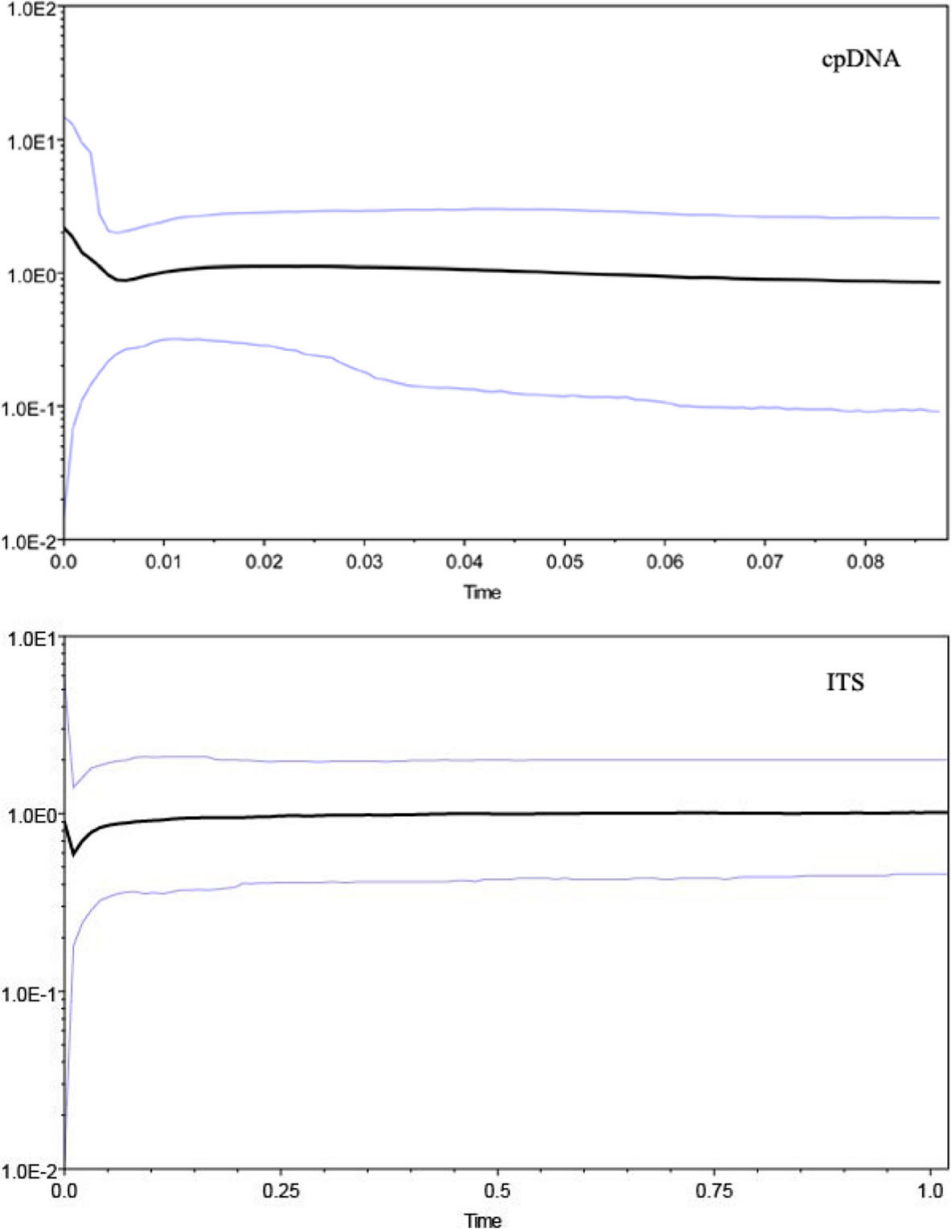
Bayesian skyline analysis based on cpDNA and nITS sequence of *Eomecon chionantha*

Both BSPs inferred from chloroplast and nrITS data revealed that *E. chionantha* experienced a recent population expansion (Fig. 3) although a weak decline in population size for nrITS occurred prior to the expansion. When the substitution rate (4.16 × 10^−9^ s/s/y) of nrITS was applied, this recent population expansion was dated to begin about 1000 years ago. While we assumed the substitution rate of the two chloroplast IGSs as 1.52 × 10^−9^ s/s/y, the population expansion event happened approximately at 6 ka.

### Present and past species distribution models

**Fig. 4:**
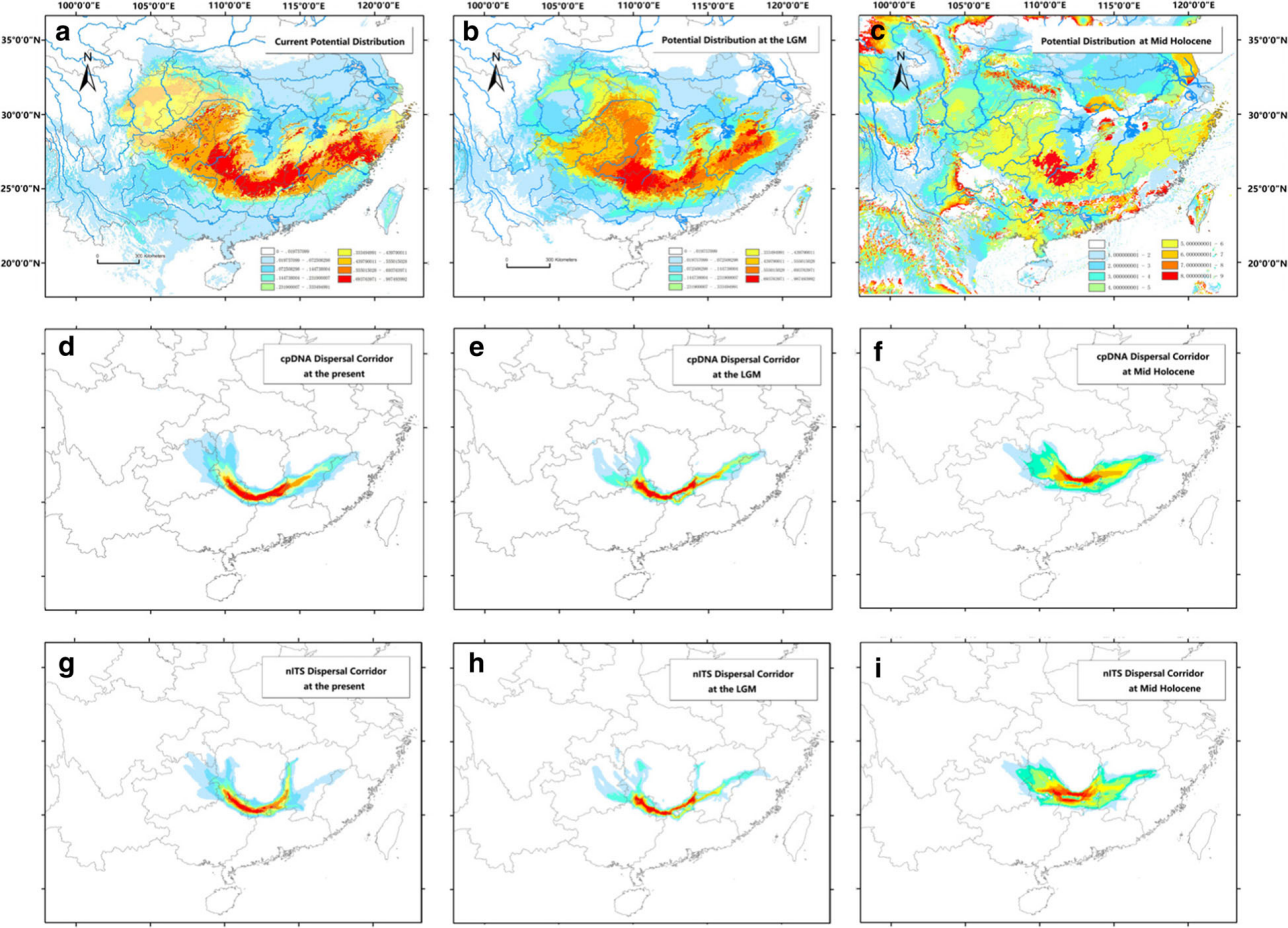
**a**, **b**, **c** Potential distribution of *Eomecon chionantha* at present, the Last Glacial Maximum (LGM) and the Mid Holocene, respectively. The climate data at LGM and MH were simulated by the Community Climate System Model (CCSM4). **d**, **e**, **f** cpDNA dispersal corridor of *E. chionantha* at the present, the LGM and the MH, respectively. **g**, **h**, **i** nITS dispersal corridor of *E. chionantha* at the present, the LGM and the MH, respectively. Warmer color depicts higher population connectivity

The AUC value for the current potential distribution of *E. chionantha* was high (0.987), indicating good predictive model performance. The predicted distribution of the species under current conditions was generally similar to its actual distribution, with a potentially continuous range from Wuyi Mountains through Nanling Mountains to Yungui Plateau (Fig. 4a). Notably, the LGM potential range of *E. chionantha* was similar to the present one, thus maintaining the connectivity between the three geographic units. The only exception was a moderate contraction at the northeast and northwest tips compared to the present potential range (Fig. 4b). The MH potential range was much larger but more fragmented than the present and the LGM ones. The most suitable habitats in the Nanling Mountains obviously contracted and somewhat moved to the north at the MH (Fig. 4c).

### Population connectivity

Both cpDNA and nrITS dispersal maps generated by the method of Chan et al. [25] showed that the most probable dispersal corridor at the LGM and the present (the areas with the hottest color) largely coincided with the Nanling Mountains, slightly extending to the Wuyi Mountains on cpDNA dispersal maps for both the present and LGM (Fig. 4d, e) or to the Luoxiao Mountains on nrITS present dispersal map (Fig. 4g). The most probable dispersal corridor at the MH slightly moved to the north edge of the Nanling Mountains (Fig. 4f, i), and the population connection in the east on nrITS dispersal map was weak (Fig. 4i).

## Discussion

### Genetic diversity and phylogeographic structure of *Eomecon chionantha*

No matter what molecular markers (chloroplast DNA sequences, nrITS sequences, nuclear microsatellites) were used, this study was not able to find obvious phylogeographic structure in *E. chionantha* (i.e., gene genealogies or genetic clusters not associated with geography), although genetic differentiation is quite high. This finding is at odds with the pronounced phylogeographic structure reported for most plant species in subtropical China that probably resulted from long-term survival in multiple Quaternary refugia and limited inter/postglacial expansions [15, 16, 53, 54]. According to recent studies, however, some plant species in this region may have experienced strong inter/postglacial range expansions, resulting in the lack of phylogeographic structure (*Sargentodoxa cuneata* [19]; *Castanopsis tibetana* and *Machilus thunbergii* [55]; *Loropetalum chinense* [56]; *Cyclocarya paliurus*, [57]). Theoretically, phylogeographic structure often arises as a consequence of restricted gene flow among populations and genetic drift within populations over relatively long periods of time [34, 58]. Although temporary genetic differentiation can arise quickly at the leading edge during range expansion due to different founders in patches [59], gene flow mediated by subsequent expanding populations may bring distantly related alleles into the same population and thus erode phylogeographic structure [34, 60]. Modelling and simulations also showed that rapid range expansion (such as leptokurtic dispersal) can produce large areas of homozygosity and these homogeneous areas may persist in space and increase with time [61]. As exemplified, Petit et al. [62] found that newly colonized populations of forest trees in north Europe displayed much weaker phylogeographic structure than those in southern glacial refugia. In this study, the demographic inferences suggest a strong (mainly northward) range expansion from putative southern refugia (located in Nanling Mountains) that could have happened at ca. 1000 to 6000 years ago, although we should caution the wide confidence in the BPS analyses. These expansions, however, could have been preceded by slight population decreases starting around the LGM (ca. 21,000 yr. BP) (Fig. 3). Therefore, it is most likely that rapid postglacial range expansion may be one of the causal factors for the lack of phylogeographic structure in *E. chionantha*. This expansion, however, occurred relatively late (starting about 6000 yr. BP), probably because the fully subtropical vegetation did not return to the Nanling Mountains and also the current range of *E. chionantha* at least until the onset of the Holocene Optimum, ca. 8000 yr. BP [63–65].

Even if *E. chionantha* have experienced an extensive northward expansion, the lack of range-wide phylogeographic structure remains elusive. In the well-studied Europe, for example, the southern range of plant species have often displayed pronounced phylogeographic structure due to long-term isolation among three peninsular glacial refugia (Iberian, Italian, and the Balkan Peninsulas) [62, 66, 67]. However, subtropical China and Europe have rather distinct physiographical characteristics that may have differential effects on phylogeographic structure. To the south of Europe, the Mediterranean Sea formed an insurmountable barrier and the three isolated peninsulas were the “last-stop” for most retreating populations during the glacial periods [68]. In contrast, subtropical China is a continuous landmass without being interrupted by seas, and has no major geographic barriers because the mountains of southern China are usually below 1500 m. The retreat to the south of many subtropical elements (such as *E. chionantha*) at the glacial periods would have ended at the Nanling Mountains, where they would have found suitable habitats. At the same time, the east-west orientation of such mountains would have allowed the connection of these refugial populations, thus lowering population differentiation. Our dispersal corridor maps based on both chloroplast and nuclear ITS data are rather coincident, both revealing considerable levels of connectivity across the Nanling Mountains. It can be concluded, therefore, that strong population connectivity maintained by the Nanling Mountains during the late Quaternary may have contributed, at least in part, to the lack of phylogeographic structure in *E. chionantha.*

The existence of cryptic refugia beyond the southern major refugia can also contribute to phylogeographic structure of plant species [62, 69]. Based on our phylogeographic data, there may be some cryptic refugia for *E. chionantha* beyond the Nanling Mountains, because populations P1, P2, P8 and P22 possess multiple and/or unique chloroplast or/and ITS haplotypes/ribotypes (and also show relatively high levels of microsatellite diversity; Table 1). However, as these haplotypes rarely occur in other populations, these populations may have contributed little to the overall genetic component of *E. chionantha*, thus having limited effects on the overall phylogeographic structure.

### The dual functions of the Nanling Mountains

Mountains provide a diverse range of thermal, hydric and edaphic conditions that would have allowed mesic habitats to be present even during the coldest, most arid phases of the glacial periods of the Pleistocene (i.e. glacial maxima); mountains, therefore, are likely to have acted as refugia for many species in both temperate and tropical regions [67, 70, 71]. In two excellent reviews of plant phylogeography in China by Qiu et al. [16] and Liu et al. [17], the Nanling Mountains are regarded as one of the major refugia in China. In this study, SDMs at the LGM, MH, and the present indicate that the habitats throughout the late Quaternary within this mountain range were suitable for the survival of *E. chionantha*. In addition, we found two ancestral (H1 and H2) and one private chlorotypes (H15) in the mountain range, further suggesting that the mountain range was also the refugial area for *E. chionantha*.

However, the number of haplotypes and genetic diversity hold by the Nanling Mountains’ populations are relatively low (only two populations within the Nanling mountains, P12 and P19, have *H*_E_ values above the mean for the species, 0.512; Table 1), contradicting the prediction that glacial refugia always harbor a large fraction of the intraspecific biodiversity ([67–69, 72]; but see [62]). Two factors may account for the relatively low genetic diversity in populations of the Nanling Mountains compared to other parts of *E. chionantha*’s range. First, populations in refugia are not necessarily the highest in genetic diversity because southern or ‘rear-edge’ populations may have lost diversity through processes such as genetic drift and diminished gene flow. For example, refugial southern populations in Europe, albeit genetically unique, were relatively impoverished in their intrapopulation genetic diveristy due to isolation in small populations [62, 73]. Second, the existence of cryptic refugia further north (see above) and/or the meeting of genetically divergent migratory routes (i.e., the ‘melting pot’ effect; [62, 74]) may also explain why some northern populations show very high levels of genetic diversity (e.g. populations P8, P9 and P14 show *H*_E_ values even higher than 0.700; Table 1).

Compared with the refugial function, the corridor role of the Nanling Mountains has only been alluded to in early literature [16, 18]. From the results of the present study, we may conclude that the population connectivity is a major cause for the lack of phylogeographic structure in *E. chionantha*. At least two factors could explain why the Nanling Mountains served as a major dispersal corridor for plant species during the late Quaternary in addition to its role as refugium. First, the Nanling Mountains is the only mountain range in southern China that shows an east-west direction; With such orientation, the Nanling Mountains enabled the connection between Yungui Plateau, the main plateau in southwestern China, and Wuyi Mountains, the main mountain range of East China. Indeed, the connection of the three main orographic units of southern China is shown not only by the maps of population connectivity of *E. chionantha,* but also by the SDMs of our previously investigated plants (*Castanopsis tibetana*, *Machilus thunbergii*, and *Schima superba*, Fig. 4; [55]). This connection allowed plants to have several recolonization routes from the Nanling glacial refugium (summarized in [19], using *Sargentodoxa cuneata* as a case-study): (i) one towards the north-west through the Yungui Plateau; (ii) another towards the east through Wuyi Mountains, and (iii) a third route through Luoxiao Mountains.

Although plains might also be utilized by plants as dispersal corridors, mountains were much more effective for plant dispersal because the climate at low elevations was very harsh (cold and arid) during the LGM [75], causing the range fragmentation of moist forest species into residual populations within mountains where the diverse habitats may facilitate their dispersal. For example, populations of *Tsuga chinensis* in southeast China separated by the Middle-Lower Yangtze Plains are distinct in genetic composition; however, those within the Nanling Mountains are much more genetically homogeneous [76]. This pattern was also observed in *Tapiscia sinensis* [77]. In contrast, mountains were relatively moist due to orographic rainfall during the glacial periods [5] and thus facilitated plant dispersal.

In addition to the orientation, the geographic location of Nanling Mountains would have also accounted for its dual role as refugium and dispersal corridor. The mountain range is located at relatively low latitude (24–26°N), the climate in this area was warm enough (mean annual temperature only 4 °C lower than the present [78]) for the persistence of plant species. More importantly, the Nanling Mountains received almost the same annual precipitation at the LGM compared to the present (the reduction was below 10%), as occurred for most of the latitudes of China located below 28°N [78]. The amplitude of precipitation is especially important for plant species like *E. chionantha* that dwells in moist forests in subtropical China, because they are adapted to the humid climate dominated by the East Asian Monsoon.

## Conclusions

We detected a lack of phylogeographic structure in a forest-dwelling herb, *Eomecon chionantha*, in subtropical China using three kinds of molecular markers. Such a phylogeographic pattern may be caused by the combined effects of a late (but rapid) postglacial expansion refugia located in the Nanling Mountains and strong population connectivity mediated by this mountain range. The hypothesis of the dual role of the Nanling Mountains provides a novel perspective for understanding the phylogeographic pattern of organisms in subtropical China. The results of this study further justify the importance of conserving the natural vegetation of the Nanling Mountains, that constitute today one of the main biodiversity hotspots in China.

## Additional files

Additional file 1: PCR primers of two chloroplast intergenic spacers and nuclear ribosomal internal transcribed spacer in *Eomecon chionantha*. (PDF 73 kb)

Additional file 2: **a**. The mean log-likelihood for each value of *K*, [ln Pr(X|K)], and Δ*K* in STRUCTURE analysis on nSSR data of *Eomecon chionantha*. **b**. DIC as a function of Kmax for nSSR in TESS analysis. (PDF 110 kb)

## Abbreviations

BSP: Bayesian skyline plots
CCSM: Community Climate System Model
Chlorotype: Chloroplast haplotype
CVH: Chinese Virtual Herbarium
DIC: Deviance Information Criterion
ESS: Effective sample sizes
HWE: Hardy-Weinberg Equilibrium
IGS: Intergenic spacers
LCP: Least cost paths
LD: Linkage disequilibrium
LGM: Last Glacial Maximum
MH: Mid-Holocene
nrITS: nuclear ribosomal internal transcribed spacer
nSSR: nuclear simple sequence repeat
SDM: Species distribution modelling
