# Supplementary material for: Phylogeography of Eomecon chionantha in subtropical China: the dual roles of the Nanling Mountains as a glacial refugium and a dispersal corridor
Source: BMC Evol Biol. 2018 Feb 9;18:20. doi: 10.1186/s12862-017-1093-x (PMC5807764; doi:10.1186/s12862-017-1093-x)
Supplement: Supplementary file 1 — PCR primers of two chloroplast intergenic spacers and nuclear ribosomal internal transcribed spacer in Eomecon chionantha. (PDF 73 kb) [file 12862_2017_1093_MOESM1_ESM.pdf]

Additional file 1 PCR primers of two chloroplast intergenic spacers and nuclear ribosomal internal transcribed spacer in *Eomecon chionantha*

	Locus	Primer sequences(5'–3')	Tm (ºC)	Ref/Accession No.
cpDNA	rbcL	ACACCAGCTTTGAATCCAAC	50	[28]
	atpB	AGAACCAGA AGTAGTAGGAT		
	trnC	CACCCRGATTYGAACTGGGG	50	[29]
	rpoB	CKACAAAAYCCYTCRAATTG		
ITS	5PF	GGAAGGAGAAGTCGTAACAAGG	53	[30]
	8PR	CACGCTTCTCCAGACTACA		
