# Supplementary material for: Phylogeography of Eomecon chionantha in subtropical China: the dual roles of the Nanling Mountains as a glacial refugium and a dispersal corridor
Source: BMC Evol Biol. 2018 Feb 9;18:20. doi: 10.1186/s12862-017-1093-x (PMC5807764; doi:10.1186/s12862-017-1093-x)
Supplement: Supplementary file 2 — a. The mean log-likelihood for each value of K, [ln Pr(X|K)], and ΔK in STRUCTURE analysis on nSSR data of Eomecon chionantha. b. DIC as a function of Kmax for nSSR in TESS analysis. (PDF 110 kb) [file 12862_2017_1093_MOESM2_ESM.pdf]

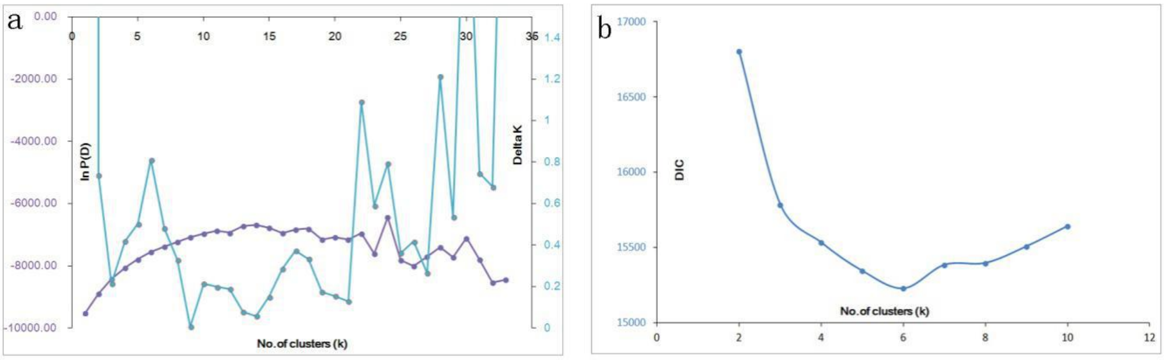

Additional file 2 a. The mean log-likelihood for each value of *K*, [ln Pr(X|K)], and ΔK in STRUCTURE analysis on nSSR data of *Eomecon chionantha.* **b.** DIC as a function of *K*max for nSSR in TESS analysis.
